# Agranulocytosis Associated with Topiramate: A Case Report and Review of Published Cases

**DOI:** 10.1155/2018/5846398

**Published:** 2018-01-10

**Authors:** Saroj Lohani, Niranjan Tachamo, Salik Nazir, Anthony Donato

**Affiliations:** ^1^Internal Medicine Residency, Reading Hospital, West Reading, PA, USA; ^2^Sidney Kimmel Medical College, Thomas Jefferson University, Philadelphia, PA, USA

## Abstract

A 41-year-old female presented to the hospital with sore throat and shortness of breath. She was hypoxic with an oxygen saturation of 87% in room air. Physical examination revealed swollen uvula with exudates. She had been started on topiramate for treatment of migraine few months ago. The dose of topiramate was increased to 100 mg twice daily 2 weeks ago. Complete blood count revealed an absolute neutrophil count (ANC) of 8 c/mm^3^. She was intubated and started on broad-spectrum antibiotics. She was transferred to our hospital on the fifth day of hospitalization. On arrival, her absolute neutrophil count was 10 c/mm^3^. Her agranulocytosis was attributed to topiramate after ruling out other possible causes. ANC improved after topiramate was stopped. ANC increased to 1000 after 5 days of stopping topiramate. We also reviewed published cases of topiramate-associated agranulocytosis. Agranulocytosis is a rare side effect of topiramate, and only 3 case reports have been published so far. In all cases, agranulocytosis developed after months of topiramate therapy and when dose was increased to 200 mg daily suggesting a dose-dependent effect. Next steps would be further research on the pathogenesis of agranulocytosis associated with topiramate and creation of registry for data synthesis.

## 1. Introduction

Topiramate is a novel anticonvulsant with multiple mechanisms of action including inhibition of voltage-activated sodium and calcium channels, augmentation of GABA receptors, and inhibition of carbonic anhydrase [1]. It is used to treat focal as well as generalized seizures. In addition, it is FDA approved for migraine prophylaxis and weight loss pharmacotherapy in combination with phentermine [1]. The main side effects of topiramate include cognitive slowing, word-finding difficulty, and impaired executive function [2]. Other common adverse effects are sedation and weight loss [2]. Agranulocytosis is severe reduction in neutrophil count less than 500 cells/mm [3, 4]. Agranulocytosis is drug induced in 70% of cases [5]. Some of the commonly associated drugs include clozapine, diclofenac, carbamazepine, vancomycin, trimethoprim/sulfamethoxazole, beta-lactam antibiotics, levamisole, and ticlopidine [5]. Agranulocytosis is a rare side effect of topiramate. In our article, we describe a case of agranulocytosis associated with topiramate and then review cases of agranulocytosis or leucopenia associated with topiramate.

## 2. Case Report

A 41-year-old female with a past medical history of Sjogren syndrome, asthma, migraines, and generalized seizures well controlled with medications was admitted to the intensive care unit after transfer from an outside hospital. She had presented to the outside hospital 5 days ago with sore throat, trouble speaking, and shortness of breath. Her family noticed that, over the last several days, she had been more confused and brought her to the emergency department for evaluation. On presentation, she was hypoxic with an oxygen saturation level of 87% in room air, temperature of 101.2 F, pulse of 110/minute, and blood pressure of 100/60 mmHg. Physical examination revealed swollen uvula without exudates. Because of her mental status and hypoxia, she was intubated and transferred to the outside hospital's intensive care unit for further care. The rest of the physical examination was within normal limits. On review of her medications, it was found that she had been treated with Plaquenil on an outpatient setting which was discontinued a month ago. She was started on topiramate for treatment of migraine by her neurologist few months ago. The dose of the topiramate had been increased to 100 mg twice a day 2 weeks ago. Rest of her medications included tramadol as needed and an albuterol inhaler. Complete blood count revealed an absolute neutrophil count (ANC) of 8 c/mm^3^. Blood and throat cultures were sent, and she was started on broad-spectrum antibiotics, including vancomycin, piperacillin-tazobactam, and clindamycin. *Streptococcus pyogenes* and *Hemophilus influenzae* were isolated from throat, and her blood culture was found to be positive for *Escherichia coli*.

When she arrived at our hospital on her hospital day 5, she was still intubated. Her ANC was 10 c/mm^3^. Labs were significant for negative polymerase chain reaction (PCR) for Epstein–Barr virus (EBV), cytomegalovirus (CMV), human immunodeficiency virus (HIV), hepatitis B virus (HBV), and hepatitis C virus (HCV). The vitamin B12 level was 600 ng/L (normal 180–914 ng/L). The folate level in blood was 12 ng/mL (normal 2–20 ng/mL). Flow cytometry of peripheral blood leucocytes showed T-cell population (about 50% of the cells analyzed) with no aberrant loss or aberrant expression of T-cell markers, a B-cell population (about 45% of the cells analyzed) that was negative for CD5 and CD10, and no surface light-chain restriction. The analyzed cells were negative for CD34, CD13, and CD33. Hematology services was consulted which did not think her agranulocytosis was due to primary bone marrow problem and recommended no need for bone marrow biopsy. Topiramate had been held from hospital day 1. Broad-spectrum antibiotics were continued. She also received 1 dose of granulocyte-monocyte colony-stimulating factor (GM CSF) on hospital day 6. Her neutrophil count started improving, and ANC was 1000 on the eleventh day of hospitalization.

## 3. Discussion

In our case, the probability of causal relationship of agranulocytosis and topiramate was assessed by the WHO-UMC algorithm [6]. Because the low numbers of ANC did return towards normal levels under continuous treatment, the probablility that the agranulocytosis related to topiramate can be rated only as probable.

In spite of the lack of evidence of efficacy in drug-induced neutropenia [7], granulocyte-monocyte colony-stimulating factor (GM CSF) is used empirically at doses of 5 mcg/kg per day. Treatment with GM CSF can be discontinued when the total white blood cell count exceeds 10,000/microL [8]. In our case, it was not clear why she received a single injection of GM CSF rather than daily injections.

Search done for cases of agranulocytosis/neutropenia/leucopenia associated with topiramate in major databases MEDLINE, Embase, and Cochrane Library revealed three case reports [9–11].

In the case reported by Minakawa et al. [10], a 40-year-old male with intractable frontal lobe epilepsy was started on topiramate 100 mg daily, eventually increased to 200 mg/day over 2 weeks. Other concurrent medications included phenytoin and acetazolamide. He was found to have an absolute neutrophil count of 440/mm^3^ on day 38 of topiramate therapy. It was not mentioned whether the patient developed any symptoms or signs from agranulocytosis. Topiramate was decreased to 100 mg/day and discontinued after a week of agranulocytosis. He was on phenytoin during the entire period. No further tests to rule out possible causes of agranulocytosis were done as there was no reduction in hemoglobin and platelets, and agranulocytosis resolved 5 days after stopping topiramate.

Behar and Schaller [9] reported a case of a 28-year-old male with a past medical history of bipolar disorder on clozapine therapy who had topiramate added as a mood stabilizer after the patient's mother was concerned about his weight gain on clozapine therapy. His white blood cell (WBC) count had been stable on clozapine for 2 years. Topiramate was added 25 mg twice a day for 1 week, 50 mg twice a day for 1 week, 100 mg twice a day for 1 week, and then 200 mg twice a day. He was followed with complete blood count every 2 weeks. He developed leucopenia (WBC count of less than 4000/mm^3^) after 1 week of topiramate 200 mg twice a day. His WBC count was 3700/mm^3^ after a week. Clozapine was stopped at that time. His WBC dropped to 3700/mm^3^ on the seventeenth day and 3100/mm^3^ on day 22 of topiramate therapy of 200 mg twice daily. He started having manic symptoms after clozapine was stopped. His family withdrew objection to weight gain, and topiramate was stopped. He was restarted on clozapine. His WBC count was 11,500/mm^3^ a week off topiramate and on clozapine. His manic symptoms resolved.

Sharma et al. [11] reported a case of a 23-year-old male with diagnosis of paranoid schizophrenia on clozapine therapy at a dose of 300 mg daily. He was started on topiramate due to concern for weight gain on clozapine therapy. Topiramate was started at 25 mg daily for a week, 50 mg daily for 2 weeks, and then 100 mg daily. He continued clozapine 300 mg/day and topiramate 100 mg/day for eight months during which time his white cell count remained under normal limits. After eight months, topiramate was increased to 200 mg per day as he was still gaining weight. The patient's dose of clozapine was not changed. On the 57th day of therapy, with topiramate 200 mg daily along with clozapine 300 mg/day, his absolute neutrophil count was 1200/mm^3^. Clozapine was discontinued. It was not mentioned whether he developed any clinical features from reduction in neutrophils. After 2 days of stopping clozapine, topiramate was tapered off. He was started on olanzapine and lithium therapy. ANC returned to normal limits. It was not clear how long it took for ANC to return to normal after stopping topiramate. This case is less clearly related to topiramate as clozapine also may cause agranulocytosis.

Agranulocytosis is a rarely reported side effect of topiramate, and its true incidence is unknown. Few cases have been reported in the medical literature. In a systematic review published by Andersohn et al. [4], 980 cases of nonchemotherapy drug-induced agranulocytosis from 1966 to 2006 were reported. There was not a single case of topiramate-associated agranulocytosis in the systematic review. However, sulfonamides including carbonic anhydrase inhibitors to which topiramate also belongs to are among the most commonly reported drugs for causing hematological disorders [12].

Drug-induced agranulocytosis can occur via multiple mechanisms. Two of the most commonly cited mechanisms include immunogenic effects (via generation of an oxidative metabolite that stimulates T cells to attack the marrow) and direct toxicity of the drug to the bone marrow [7]. The pathogenesis of agranulocytosis associated with topiramate is not clear. In cases of immune-mediated neutropenia/agranulocytosis, neutrophil count usually drops rapidly within 5 to 7 days of drug administration [3]. Immune-mediated agranulocytosis is often accompanied by rash and fever as seen in cases of agranulocytosis associated with beta-lactam antibiotics, which was not seen in our case or any of the reviewed cases [3]. Topiramate (sulfamate) is structurally similar to sulfones (dapsone) and sulfonamides (acetazolamide, sulfonylureas, and sulfamethoxazole). Dapsone causes agranulocytosis due to the toxic action of its metabolites against neutrophils and bone marrow [13]. In our case as well as in the other reported cases, reduction in neutrophil count was detected after months of topiramate therapy. In the reported cases, neutrophil count decreased when the dose of topiramate was increased to 200 mg daily, suggesting a dose-dependent effect. No reduction in neutrophil count was seen with doses less than 200 mg daily [11]. These all suggest that topiramate-associated reduction in neutrophil count is most likely secondary to toxic effects of the drug. However, further research is required in future to elucidate the mechanism of topiramate-associated agranulocytosis.

## 4. Conclusion

Topiramate is a broad-spectrum novel anticonvulsant used for seizure treatment, migraine prophylaxis, and weight loss. Agranulocytosis/neutropenia is a rare side effect of topiramate and is limited to few case reports. The pathogenesis of reduction in neutrophil count associated with topiramate is not entirely known. In our case as well as in other published cases, reduction in neutrophil count occurred after months of topiramate therapy and at doses greater than or equal to 200 mg daily. In all cases, neutrophil count returned to normal after topiramate was stopped. Next steps would be further research on the pathogenesis of agranulocytosis associated with topiramate and creation of registry for data synthesis.
